# Eastern Cooperative Oncology Group classification and its application in surgical treatment planning of epithelial ovarian cancer

**DOI:** 10.3389/fonc.2026.1869660

**Published:** 2026-07-09

**Authors:** Siiri Karjalainen, Auni Lindgren, Merja Kokki, Julia Heikkinen, Maarit Anttila

**Affiliations:** 1 School of Medicine, Faculty of Health Sciences, University of Eastern Finland, Kuopio, Finland; 2 Obstetrics and Gynecology, Kuopio University Hospital, Kuopio, Finland; 3 Anaesthesiology and Intensive Care Kuopio University Hospital, Kuopio, Finland

**Keywords:** complications, length of stay, ovarian cancer, physical fitness, surgical treatment, survival

## Abstract

**Introduction:**

Patients’ physical performance status reflects the body’s recovery reserve against physiological stressors like surgery. The aim of our study was to evaluate the association of clinical factors, primarily Eastern Cooperative Oncology Group (ECOG) classification, with patient performance status and their use in clinical decision-making for surgical treatment of epithelial ovarian cancer (EOC).

**Materials and methods:**

We retrospectively enrolled 228 patients with EOC who underwent debulking surgery (either primary or interval setting) in Kuopio University Hospital during the years 2019–2022. Physical fitness according to the ECOG scale and other clinical parameters were collected from the patients’ hospital records. The association of ECOG with other variables was analyzed using Kruskal–Wallis test. Univariate survival analysis was conducted with Kaplan–Meier and multivariate survival analysis was carried out with Cox regression.

**Results:**

From 228 women who underwent debulking surgery, 129 (56.6%) patients belonged to ECOG 0, 76 (33%) patients were part of ECOG 1, and only 23 (10.1%) belonged to ECOG 2–4. Age, self-reported walking distance, stair climbing capacity, ASA classification, albumin, prealbumin, and number of comorbidities were statistically significantly associated with ECOG groups. Interestingly, ECOG was not associated with complications or length of hospital stay. Instead, lower ECOG predicted higher survival rates (ECOG 0 *p* = 0.003, ECOG 1 *p* = 0.005, HR = 3.785, ECOG 2–4 *p* = 0.002, HR = 10.231).

**Discussion and conclusion:**

It is important to make precise patient selection for operative treatment in EOC. In our study, ECOG classification emerged as a useful tool in evaluating patients’ physical status.

## 1 Introduction

Epithelial ovarian cancers (EOCs) are the seventh most common cancer among women globally (1). EOC is the most lethal gynecological cancer as its 5-year survival is only 46%. EOC is usually diagnosed (75% of cases) in advanced stages, which partly explains its lethality. The nature of the disease is asymptomatic. If patients present symptoms, they are usually non-specific, such as abdominal bloating, early satiety, nausea, change in bowel function, urinary symptoms, fatigue, and weight loss.

The diagnosis consists of pelvic ultrasound, tumor marker cancer antigen 125 (CA125) measurement, and further imaging including chest and abdomen computed tomography (CT) or magnetic resonance imaging (MRI). Histological samples are also crucial for diagnosis and for deciding the appropriate course of treatment and prognosis. Precise staging requires a staging surgery with total abdominal hysterectomy, bilateral salpingo-oophorectomy, omentectomy, inspection of peritoneal surfaces, and biopsy/removal of suspicious areas and para-aortic and pelvic lymph node dissection if needed. The staging operation will determine the FIGO (International Federation of Gynecology and Obstetrics) surgical stage.

The standard treatment for EOC is primary debulking surgery (PDS) followed by chemotherapy in advanced stages. PDS is the preferred route for patients with FIGO stage up to IIIC and small (<5 cm) metastases, whereas stage IV is commonly treated with neoadjuvant chemotherapy (NACT) and interval debulking surgery (IDS). The amount of residual disease after a cytoreductive surgery is an independent and the most important prognostic factor for survival (1–3). In order to achieve this goal, expanded procedures such as peritonectomies, diaphragmatic peritonectomies, resection of subcapsular liver metastases, splenectomy, bowel resection, and resection of extra-abdominal metastases are performed (2).

A comprehensive evaluation of patient characteristics is essential in deciding the patient’s suitability for a debulking surgery and the appropriate time for it (4). Complex procedures can lead to severe complications, prolonged recovery periods, and delays in the initiation of adjuvant chemotherapy. Therefore, the patient’s age, physical reserve, and the type and stage of cancer should be taken into consideration when planning the treatment. It is known that the patient’s physical status reflects on the body’s recovery reserve after physiological stressors like surgery. One possible method to determine the performance status of patients with EOC is Eastern Cooperative Oncology Group (ECOG) classification. ECOG classification was created to assess the suitability of patients with cancer for withstanding chemotherapy treatment (6). To this day, it is primarily used in drug trials, although its use has expanded to assess candidacy for surgical interventions as well (2, 4, 7, 8).

The aim of our study was to evaluate the association of different clinical factors with patient performance status, determined by ECOG classification and whether the use of this simple evaluating tool can help clinicians plan the appropriate timing and course of surgical treatment for patients with EOC taking into consideration postoperative complications, the length of hospital stay (LOS), and survival.

## 2 Materials and methods

### 2.1 Participants

This retrospective observational study was performed in Kuopio University Hospital and had an institutional permit (695/13.00/2023).

**Figure 1:**
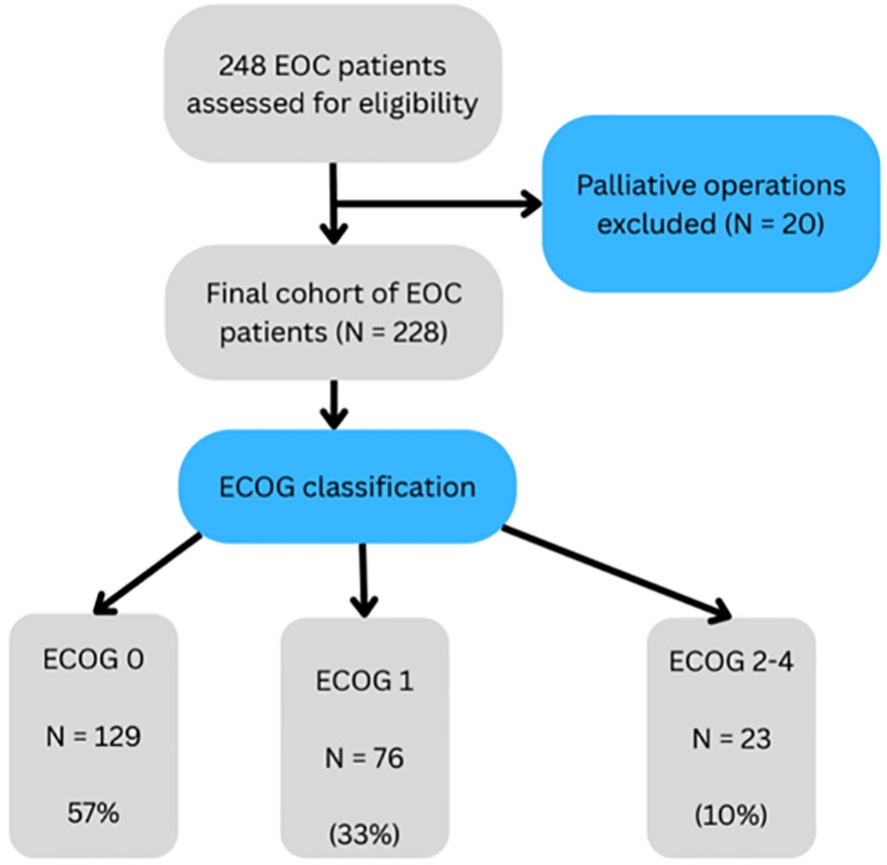
Study cohort diagram. ECOG, Eastern Cooperative Oncology Group scale.

Our patient data were gathered from the hospital’s patient records. Inclusion criteria were newly diagnosed patient with EOC who underwent primary surgery at Kuopio University Hospital during the years 2019–2022. Exclusion criteria were only chemotherapy for primary treatment. A total of 248 patients were included in the cohort, and from those, 20 palliative operations were excluded due to different goals of the operation. The total number of 228 patients was included in the final analysis. The data were categorized by ECOG performance status scale into three groups: ECOG 0, ECOG 1, and ECOG 2–4 (see **Figure 1**).

### 2.2 Evaluation of performance status

The operating gynecological oncologist determined patients’ ECOG status in preoperative outpatient clinic visit. The criteria for determining the ECOG performance status were clearly defined, and in cases of uncertainty, the patient’s ECOG status was discussed collaboratively with an anesthesiologist (see **Table 1**) (6).

**Table 1:** ECOG performance status classification.

Grade	ECOG performance status
0	Fully active, pre-disease performance
1	Disease restricts strenuous activity but ambulatory and can carry out light work
2	Ambulatory and can vary out all self-care, unable to perform work activities but up and about >50% of waking hours
3	Capable of only limited self-care, confined to bed or chair >50% of waking hours
4	Completely disabled, unable to carry out self-care and totally confined to bed or chair
5	Dead

Self-reported walking and stair climbing capacity were determined preoperatively by a questionnaire (9). Walking capacity was divided into two groups: those able to walk ≤500 m and >500 m. Staircases were divided into three groups: 0–1, 2–3, and >3 staircases. American Society of Anesthesiologists (ASA) classification (5), body mass index (BMI), and comorbidities were also considered in the evaluation of physical status. The patient was considered to have multimorbidity at ≥2 comorbidities (10). Preliminary FIGO stage was finalized after the staging procedure. FIGO stage was divided into four groups: I, II, III, and IV.

Patients’ nutritional status was assessed via serum albumin and prealbumin measurements. A total of 214 (93.9%) patients underwent a laparotomy, and 14 (6.1%) patients underwent a laparoscopic operation. The debulking surgery was considered radical if it included a bowel resection and/or procedures in the upper abdominal cavity, like diaphragm stripping. A total of 102 (44.7%) operations were radical and 126 (55.3%) were standard. The amount of macroscopic residual disease was assessed during surgery. A total of 158 (69.3%) had no visible residual disease, 32 (14.0%) had <1 cm of residual disease, and 36 (15.8%) had >1 cm of residual disease.

### 2.3 Statistical methods

Patient data were analyzed using SPSS (IBM SPSS Statistics Version 29.0). For overview, the data were categorized by ECOG groups (ECOG 0, ECOG 1, and ECOG 2–4), and the association of ECOG classification with other clinical variables was analyzed using Kruskal–Wallis test for continuous variables and cross-tabulation for categorized variables. Data normality check was performed with the explore function.

Complications were assessed with Clavien–Dindo (CD) classification. CD classification was divided into three categories: CD 0 (no complications), CD 1–3a (mild complications), and CD ≥3b (serious complications). To identify factors associated with complications, binary logistic regression was used. CD classification was dichotomized (CD < 3b and CD ≥ 3b) to conduct this analysis.

The association of physical status, FIGO stage, residual disease, and comorbidity with survival was examined with Kaplan–Meier. Albumin was categorized into three groups: >35, 30–35, and <30 g/L. Prealbumin was also divided into three groups: >0.18, 0.10–0.18, and <0.10 g/L (11). The FIGO stage was dichotomized (FIGO 1–2 and 3–4) for survival analysis. Statistically significant results in univariate survival analysis were included in the multivariate Cox regression model.

The association of performance-describing factors and surgery-related factors with LOS was analyzed first with Spearman correlation followed by multivariate quantile regression analysis. ECOG scale, ASA classification, self-reported walking and stair climbing capacity, comorbidity, albumin and prealbumin, BMI, CD, duration of surgery, radicality of the operation, bleeding in the operation, and FIGO stage were included in the analysis.

## 3 Results

### 3.1 The association of ECOG class with other indicators of physical fitness

A total of 228 women who underwent debulking surgery for EOC were enrolled in the study. In total, 193 (84.6%) patients underwent PDS and 35 (15.4%) patients had NACT before the debulking surgery. ECOG group 0 was the largest group with 129 patients (56.6%). ECOG group 1 had 76 patients (33%) and ECOG group 2–4 had 23 patients (10.1%) (see **Tables 2**, **3**).

**Table 2:** Patient characteristics according to different ECOG groups.

Variable	ECOG 0	ECOG 1	ECOG 2–4	*P*-value
Total	*n* = 129	*n* = 76	*n* = 23	
Age (years)	64.0 (26–82)	73.0 (48–90)	71 (30–84)	<0.001
Menopause status				0.011
Pre	18 (14.0%)	1 (1.3%)	3 (13.0%)	
Post	111 (86.0%)	75 (98.7%)	20 (87%)	
BMI (kg/m^2^)	*n* = 129 (56.6%)	*n* = 76 (33.3%)	*n* = 22 (9.6%)	0.500
	26.0 (17.9–39.6)	26.9 (18.3–39.0)	27.3 (19.0–45.2)	
Walking distance				
>500 m	124 (96.1%)	45 (59.2%)	4 (17.4%)	<0.001
<500 m	4 (3.1%)	29 (38.2%)	17 (73.9%)	
Number of staircases				<0.001
>3	80 (62.0%)	17 (22.4%)	3 (13.0%)	
2–3	39 (30.2%)	32 (42.1%)	2 (8.7%)	
0–1	2 (1.6%)	23 (30.3%)	16 (69.6%)	
**ASA,** *n* (%)				<0.001
1	33 (25.6%)	1 (1.3%)	0	
2	76 (58.9%)	28 (36.8%)	1 (4.3%)	
3	20 (15.5%)	43 (56.6%)	16 (69.6%)	
4	0	4 (5.3%)	6 (26.1%)	
p-Albumin (g/L)	*n* = 127 37.0 (22.0–42.0)	*n* = 76 34.0 (17.0–42.0)	*n* = 21 31.0 (10.0–38.0)	<0.001
p-Prealbumin (g/L)	*n* = 113 0.23 (0.08–0.37)	*n* = 69 0.20 (0.08–0.35)	*n* = 17 0.20 (0.06–0.41)	0.040
Comorbidities, *n* (%)				<0.001
No	44 (34.1%)	10 (13.2%)	0	
1	43 (33.3%)	17 (22.4%)	3 (13.0%)	
≥2 diseases	42 (32.6%)	49 (64.5%)	20 (87.0%)	

Continuous variables are reported with median and range in parentheses. ECOG, Eastern Cooperative Oncology Group scale; BMI, body mass index; ASA, American Society of Anaesthesiologists.

**Table 3:** Disease and operation characteristics according to different ECOG groups.

Variable	ECOG 0	ECOG 1	ECOG 2–4	*P*-value
Total	*n* = 129	*n* = 76	*n* = 23	
FIGO				0.093
1	36 (27.9%)	10 (13.2%)	7 (30.4%)	
2	8 (6.2%)	8 (10.5%)	0	
3	46 (35.7%)	32 (42.1%)	11 (47.8%)	
4	39 (30.2%)	26 (34.2%)	4 (17.4%)	
Operation type				NS
Laparotomy	121 (93.8%)	71 (93.4%)	22 (95.7%)	
Laparoscopy	8 (6.2%)	5 (6.6%)	1 (4.3%)	
Residual disease				0.010
NVD	99 (78.0%)	47 (61.8%)	12 (52.2%)	
<1 cm	16 (12.6%)	10 (13.2%)	6 (26.1%)	
>1 cm	12 (9.4%)	19 (25.0%)	5 (21.7%)	
Treatment order				0.200
PDS	114 (88.4%)	61 (80.3%)	18 (78.3%)	
NACT	15 (11.6%)	15 (19.7%)	5 (21.7%)	
Clavien–Dindo				0.093
0	35 (27.1%)	29 (38.2%)	12 (52.2%)	
1–3a	75 (58.1%)	38 (50.0%)	7 (30.4%)	
≥3b	19 (14.7%)	9 (11.8%)	4 (17.4%)	
Radicality of the operation				0.403
Non-radical/standard	67 (51.9%)	39 (51.3%)	13 (56.5%)	
Radical operation	62 (48.1%)	34 (44.7%)	6 (26.1%)	
Histology				NS
High-grade serous	87 (67.4%)	58 (76.3%)	13 (56.5%)	
Low-grade serous	10 (7.8%)	0	1 (4.3%)	
Musinous	8 (6.2%)	1 (1.3%)	4 (17.4%)	
Endometrioid	12 (9.3%)	6 (7.9%)	4 (17.4%)	
Clear cell	4 (3.1%)	2 (2.6%)	0	
Other (carcinosarcoma)	5 (3.9%)	8 (10.5%)	1 (4.3%)	
Multiple types	3 (2.3%)	0	0	

Continuous variables are reported with median and range in parentheses. ECOG, Eastern Cooperative Oncology Group scale; FIGO, International Federation of Gynecological Oncology; NVD, no visible disease; PDS, primary debulking surgery; NACT, neoadjuvant chemotherapy.

The analysis revealed that age, menopausal status, self-reported walking and stair climbing, ASA classification, albumin and prealbumin, comorbidity, and residual disease were statistically significantly associated with ECOG status. Most of ECOG group 0 patients (84.5%) had ASA 1 or 2 classification whereas only 39.1% of patients in ECOG group 1 and 4.3% in ECOG group 2–4 had ASA 2 classification. Plasma albumin and prealbumin levels were higher in the lower ECOG groups, *p* < 0.001 and *p* = 0.04, respectively. In ECOG group 0, 32.6% of the patients had multimorbidity, while in ECOG group 1 and ECOG group 2–4, 64.5% and 87.0% had multimorbidity, respectively.

### 3.2 The relation of physical fitness to surgical complications

The association of mild (CD < 3b) versus severe (CD ≥ 3b) complications with ECOG classification, peri-operational bleeding, the duration of the operation (hours), comorbidity, age, BMI, albumin (<30, 30–35, and >35), prealbumin (<0.10, 0.10–0.18, and >0.18), walking capacity (0–500 m and >500 m), and stair climbing capacity (0–1, 2–3, and >3) was examined with the binary logistic regression model. According to our results, out of the clinical factors mentioned above, only multimorbidity (≥2 comorbidities) was statistically significantly (*p* = 0.048) associated with the rate of complications.

### 3.3 Physical fitness and survival

Overall survival at 1 year postoperatively was 90%, whereas overall survival at 2 and 3 years postoperatively was 83% and 75%, respectively. Overall mortality after 30 days was 1.3%, and after 90 days, it was 2.2%. **Table 4** demonstrates the effect of different clinical parameters to survival. Prealbumin and CD were not statistically significantly associated with survival.

**Table 4:** The connection of different physical, cancer-related, and surgical parameters to survival rates.

Variable	*P*-value	Survival 12 months (%)	Survival 24 months (%)	Survival 36 months (%)
Age <75	0.002	91.7	86.3	77.2
Age ≥75		81.8	72.4	60.0
ASA 1	<0.001	100	100	95.2
ASA 2		91.9	83.9	79.4
ASA 3–4		82.7	75.3	55.6
ECOG 0	<0.001	95.3	92.0	88.6
ECOG 1		83.4	72.0	54.9
ECOG 2–4		77.0	71.9	51.3
Albumin <30	0.001	78.7	62.1	51.6
Albumin 30–35		89.4	85.5	70.2
Albumin >35		93.6	89.9	82.3
Walking distance ≤500 m	0.031	87.5	82.5	62.4
Walking distance >500 m		90.2	83.9	80.0
Staircases 0–1	0.026	87.8	84.9	60.9
Staircases 2–3		87.2	77.8	67.9
Staircases >3		92.7	90.2	86.0
FIGO: 1–2	0.003	95.5	93.5	90.2
FIGO 3–4		86.6	78.4	66.4
Residual disease 0 cm	<0.001	97.5	94.2	94.2
Residual disease <1 cm		90.2	85.5	67.2
Residual disease >1 cm		67.1	49.2	25.3
Comorbidity 0	0.037	96.0	90.6	86.7
Comorbidity 1		91.6	85.3	80.2
Comorbidity ≥2		84.9	78.0	64.9

ASA, American Society of Anesthesiologists; ECOG, Eastern Cooperative Oncology Group scale; FIGO, International Federation of Gynecology and Obstetrics.

According to this univariate survival analysis, age ≥75 years, high ASA classification and ECOG scale, multimorbidity, low albumin, and low walking and stair climbing capacity significantly decrease the rate of survival.

Based on the results, age, BMI, ECOG classification, albumin, walking and stair climbing capacity, FIGO stage, residual disease, and comorbidity were included in multivariate Cox regression analysis (see **Table 5**). ASA classification was excluded due to its strong correlation with ECOG scale. Physical factors other than ECOG status did not statistically significantly impact survival.

**Table 5:** The effect of different patient characteristics to survival.

Variable	*P*-value	HR	95% CI
ECOG 0	0.002		
ECOG 1	0.006	3.425	1.413–8.307
ECOG 2–4	<0.001	10.745	2.675–43.167
Residual disease 0 cm	0.007		
Residual disease < 1 cm	0.218	1.796	0.708–4.558
Residual disease > 1 cm	0.002	4.440	1.762–11.189
Comorbidity 0	0.591		
Comorbidity 1	0.806	1.170	0.333–4.104
Comorbidity ≥2	0.398	1.635	0.523–5.112
FIGO: 1	0.256		
FIGO 2		1.811	0.650–5.047
Staircases 0–1	0.329		
Staircases 2–3	0.220	0.486	0.153–1.540
Staircases >3	0.871	1.073	0.460–2.501
Walking distance ≤500 m	0.566		
Walking distance >500 m		0.758	0.295–1.950
Albumin <30	0.562		
Albumin 30–35	0.775	1.129	0.491–2.600
Albumin >35	0.404	0.697	0.298–1.629
Age <75	0.243		
Age ≥75		1.556	0.741–3.267
BMI	0.995	1.000	0.941–1.063

ECOG, Eastern Cooperative Oncology Group scale; HR, hazard ratio; FIGO, International Federation of Gynecology and Obstetrics; BMI, body mass index.

### 3.4 The association of physical fitness with the length of hospital stay

With Spearman’s correlation, FIGO stage (*r* = 0.262, *p* = 0.0001), albumin level (*r* = −0.168, *p* = 0.018), CD classification (*r* = 0.488, *p* < 0.001), duration of the surgery (*r* = 0.294, *p* = 0.0001), radicality of the operation (*r* = 0.321, *p* < 0.001), and bleeding in the operation (*r* = 0.339, *p* < 0.001) were significantly correlated with LOS. They were included to multivariate quantile regression analysis with ECOG and walking distance. According to the results, only complications increased LOS statistically significantly; regression coefficient for CD 1–3a was 1.974 (*p* ≤ 0.001, 95% CI 1.060–2.888) and that for CD ≥ 3b was 3.408 (*p* ≤ 0.001, 95% CI 2.096–4.721). In contrast to our hypothesis, ECOG status did not associate with the LOS statistically significantly.

## 4 Discussion

Patients with ovarian cancer are a complex and heterogeneous patient group. The gold standard treatment is debulking surgery, either in a primary setting or after neoadjuvant chemotherapy combined to adjuvant platinum-based chemotherapy (1). The surgical procedure imposes significant physiological stress, necessitating prompt postoperative recovery to enable timely initiation of adjuvant chemotherapy (4). Consequently, we hypothesized that physical performance status (ECOG) would have an impact on complications, survival, and LOS.

Our patient cohort offers real-world data about the physical characteristics of patients with EOC in addition to data about the surgical treatment itself. ECOG status was associated with several clinical factors just as age, ASA classification, albumin and prealbumin levels, number of comorbidities, self-reported walking distance, and stair climbing capacity. This indicates that ECOG status is useful in evaluating surgical patients’ physical status.

Regarding the association of physical status (ECOG) with surgical complications, our hypothesis was that the patients of a higher physical capacity (low ECOG group) would have less complications. Surprisingly, ECOG classification did not associate with the odds of complications statistically significantly. Generally, in previous research, complication risk is higher in weaker physical status groups (2, 4, 7, 12, 13). Our result may be different due to the size and proportion of our patient material. The number of patients in ECOG group 2–4 is smaller than that in the other groups. The majority of ECOG group 2–4 patients were not operated radically, although difference was not statistically significant.

Patients’ diagnostical ECOG 0 status was significantly associated with better survival in our cohort, which supports pre-existing research on the effect of physical status in ovarian cancer treatment (2, 4, 7, 12, 14). Residual disease after operation has been proven to be the most important factor for survival (1–3). In our cohort, there was no statistically significant association between ECOG status and residual disease. Both ECOG and residual disease remained significant predictors for survival in multivariate Cox regression analysis. Therefore, ECOG is useful in the clinical evaluation and treatment planning of patients with EOC.

As for the LOS, according to our results, surgery-related factors, primarily complications, affect the LOS whereas physical status (ECOG status) did not. Despite this, in previous studies, malnutrition, muscle attenuation, and, therefore, reduced physical capacity prolong hospitalization, although the field of research on postoperative hospitalization of EOC surgery is scarce (12, 13, 15). The proportion of our patient cohort in addition to the lack of a wider assessment of fitness may once more result in that only the rate of complications stood out statistically significantly behind prolonged LOS. Conducting the same analysis with a larger patient cohort could offer more comprehensive insight into this matter. For instance, the studies by Sehouli et al. (2021) and Argillander et al. (2022) support this conclusion (12, 16).

Furthermore, a preoperative exercise regimen and enhanced nutrition plan could improve patients’ physical status and prognosis after treatment (17). Although the physical condition of ECOG > 2 patients does not allow them to spend most of the day upright, it is not necessarily a fixed state for everyone. Treating malnutrition and gradually increasing daily activity can lead to improvement in ECOG classification. Zebalski et al. (2024) found that preoperative prehabilitation, including an exercise regimen and nutritional support, led to functional improvement in the 6-min walk test and furthermore a shorter hospitalization, fewer postoperative complications, and better overall recovery after ovarian cancer surgery (18). Similarly, Miralpeix et al. (2022) reported that prehabilitation during neoadjuvant chemotherapy before IDS for ovarian cancer resulted in improved nutritional markers, fewer intraoperative complications, and faster postoperative recovery in their small cohort (19). These findings support the concept that patients with more advanced cancer and consequently poorer physical condition can still achieve improvements. The results of Cham et al. (2025) suggest that frailty screening is underused in ovarian cancer care and that a multidisciplinary prehabilitation approach, focusing on nutrition, physiotherapy, and psychosocial support, may benefit vulnerable patients (20). Inci et al. (2021) highlighted the significance of a poor physical condition, as measured by the frailty index, in predicting postoperative complications and worse outcomes after cytoreductive surgery, suggesting that targeted interventions are warranted (21). Lastly, McIsaac et al. (2026) conducted a home-based exercise and personal nutrition prehabilitation program for older frail patients and found that even severely frail individuals can improve their condition, although this improvement could not be directly linked to better postoperative outcomes (22).

With respect to restrictions in this study, besides the retrospective study approach, we had to evaluate patients’ nutritional status via serum albumin and prealbumin measurements due to the lack of more informative data. Evaluation of sarcopenia via CT scans would offer more insight into patients’ physical status. In the study by Aust et al. (2015), sarcopenia was linked to poorer surgical outcome and postoperative morbidity and mortality (8). Heus et al. (2021) found sarcopenia and visceral obesity to predict surgical complications (23). Furthermore, we consider the lack of more objective measurements of physical status such as walking tests and cycle ergometer tests a limitation in our study. The strength of this study is that the use of standardized assessment tool ECOG performance status is easy to use, cost-effective, and reproducible.

## 5 Conclusion

It is important to make precise patient selection for operative treatment in ovarian cancer. According to our results, ECOG classification is a useful tool in the treatment planning of patients with ovarian cancer. Although ECOG classification at the time of diagnosis was not statistically significantly associated with postoperative complications and the LOS, it was still statistically significantly associated with survival of ovarian cancer.

## Data Availability

The raw data supporting the conclusions of this article will be made available by the authors, without undue reservation.
